# Neurogenesis and pesticides: news of no new neurons

**DOI:** 10.1055/s-0044-1786853

**Published:** 2024-05-19

**Authors:** Fulvio A. Scorza, Antonio-Carlos G. de Almeida, Ana C. Fiorini, Feres Chaddad-Neto, Josef Finsterer

**Affiliations:** 1 Universidade Federal de São Paulo, Escola Paulista de Medicina, Departamento de Neurologia, Disciplina de Neurologia, São Paulo SP, Brazil.; 2 Ministério do Desenvolvimento Agrário e Agricultura Familiar, São Paulo SP, Brazil.; 3 Universidade Federal de São João del-Rei, Departamento de Engenharia de Biossistemas, Laboratório de Neurociência Experimental e Computacional, São João del-Rei MG, Brazil.; 4 Universidade Federal de São Paulo, Escola Paulista de Medicina, Departamento de Fonoaudiologia, São Paulo SP, Brazil.; 5 Pontifícia Universidade Católica de São Paulo, Programa de Estudos Pós-Graduado em Fonoaudiologia, São Paulo SP, Brazil.; 6 Universidade Federal de São Paulo, Escola Paulista de Medicina, Departamento de Neurologia e Neurocirurgia, São Paulo SP, Brazil.; 7 Hospital Beneficência Portuguesa, Unidade de Neurocirurgia, São Paulo SP, Brazil.; 8 Neurology and Neurophysiology Center, Vienna, Austria.

**Keywords:** Neurogenesis, Pesticides, Neuronal Plasticity, Brain, Neurogênese, Praguicidas, Plasticidade Neuronal, Encéfalo

## Abstract

New hippocampal neurons are continuously generated in the adult human brain. Several studies have demonstrated that the proliferation of hippocampal cells is strongly influenced by a variety of stimuli, including pesticides exposure. These effects are particularly important because neurogenesis dysregulation could be associated with the decline of neuronal and cognitive functions and the possible development of neuropsychiatric disorders.

## Resumo

Novos neurônios hipocampais são gerados continuamente no cérebro humano adulto. Vários estudos têm demonstrado que a proliferação de células do hipocampo é influenciada por uma variedade de estímulos, incluindo a exposição a pesticidas. Estes efeitos são particularmente importantes porque a desregulação da neurogênese pode estar associada ao declínio das funções neuronais e cognitivas e ao possível desenvolvimento de doenças neuropsiquiátricas.

## FROM HISTORICAL NOTES TO INTRODUCTION

The human brain is considered the most complex system and most fascinating part of our organism. The brain consists of 86 billion neurons and weighs ∼ 1.5 kg. It is responsible for our consciousness, orientation, perception, thinking, language, motor functions, sensations, emotions, and hormonal and vegetative control. 1 In fact, neurons are the basic units of the nervous system and are considered the main elements involved in the generation, transmission, and processing of cerebral information. 2 Throughout life, the human brain constantly changes and, therefore, becomes a flexible and adaptable organ. This neuroplasticity, the ability of the adult brain to change its anatomy, connectivity, and networks in response to external and internal stimuli, allows neurons to structurally reorganize and form new cells and adjust their number, morphology, and function in response to changes in the environment. 3 4 5 6 The formation of these new neurons (neurogenesis) in an already existing neuronal network is one of the most important examples of neuroplasticity 3 4 5 6 and a really fascinating process.

In the late 19th century and early 20th century, the Spanish physician and histologist Ramon y Cajal (Nobel Prize for Physiology and Medicine in 1906), identified the microscopic anatomy of the central nervous system (CNS) and postulated: “In the CNS of adults, the neuronal pathways are solid and unchangeable. All cells must die, and regeneration does not take place. Maybe science will change this law in the future.” 6 In fact, science has succeeded in relativizing this paradigm. In the first half of the 20th century, some researchers suspected the existence of a cell division process in the brains of rats after birth, 7 8 9 without specifying whether these new cells would transform into neurons. Advances in understanding neurogenesis were made following the introduction of the (autoradiographic 3H)-thymidine technique, which incorporates into the DNA (deoxyribonucleic acid) of dividing cells. Using this technique, a research group led by Joseph Altman demonstrated neurogenesis in several brain structures of young and adult rats, including the hippocampus, neocortex, and olfactory bulb. 10 11 12 The researchers argued that these new “microneurons” were involved in learning and memory processes. 13 However, since the techniques available at the time were unable to accurately show whether these cells were themselves neurons, the results demonstrated by Altman were ignored. With technological advances, the advent of electron microscopy made it possible to accurately state that cells in the hippocampus and olfactory bulbs of adult rats that incorporated (3H)-thymidine had the structural properties of neurons. 14

In addition, there were advances in the study of neurogenesis in the 90s, with the development of the synthetic analogue of thymidine, BrdU (5-chromo-3′-deoxyuridine). BrdU is taken up by cells during the cell synthesis phase (S phase of mitosis) and is, therefore, a marker for proliferating cells. Labeling of cell nuclei with BrdU can be clearly visualized by immunocytochemical techniques, without the need for autoradiography. 15 After this period, the process of neurogenesis in the adult CNS was described in several species of invertebrate and vertebrate animals, including crustaceans, reptiles, amphibians, birds, rodents, primates, and humans. 16 17 18 19 20 21 Among the neurogenic zones of the adult brain, the hippocampal formation has been extensively studied because it is involved in higher cognitive functions, mainly in memory processes and in certain affective behaviors. 22 Therefore, one of the main populations of hippocampal neurons, the granule cells of the dentate gyrus, carry the property of postnatal neurogenesis. 10 20 The majority of granule cells in the dentate gyrus are formed in the postnatal period; however, complete development of the granule cell layer occurs between approximately days 20 and 25 of life. 20 21 22 Granule cells originate from precursor cells located in the hilus of the dentate gyrus. Initially, they spread throughout the hilus and by the second postnatal week they are located in the subgranule zone of the dentate gyrus and remain mitotically active (the production of new cells was estimated at 1 neuron/2,000 existing granule cells, that is, in a mouse of 3 months, hundreds of cells are produced per day). 20 21 22 In the vast majority of organisms, including humans, this process can continue for long periods of time, probably until senescence. 23

## FACTORS REGULATING NEUROGENESIS IN THE ADULT HIPPOCAMPUS

In recent decades, it has been repeatedly demonstrated that the process of neurogenesis in the adult CNS is strongly influenced by a variety of stimuli. In 1995, American researchers showed that prenatal malnutrition caused by protein deficiency significantly alters the profile of postnatal neurogenesis in the hippocampal region in laboratory animals and that this process persists even with nutritional rehabilitation after birth. 24 Currently, several studies have confirmed the influence of nutrition and diet on modulating neurogenesis in the hippocampus. 25 In this sense, diets high in fats and/or sugars have been reported to have negative effect on neurogenesis, while diets enriched with bioactive compounds, such as polyunsaturated fatty acids and polyphenols, can induce the formation of new neurons. 25 In 1997, experimental studies showed increased neurogenesis in the hippocampal region of mice that lived in enriched environments (environments that provide cognitive, sensory, and motor stimuli) compared with mice that lived in laboratory cages. 23 Due to their importance to humans, recent studies suggest that enriched environments may increase brain activity, and serve as possible non-pharmacological approaches in the prevention and/or progression of neurodegenerative diseases. 26 At the same time, other studies have confirmed that the production and survival of new hippocampal neurons can be increased or decreased by experience. 27 It has been observed that aversive experiences (stress) decrease the production of new neurons, while enriching experiences (learning) increase the survival of new hippocampal cells. 27 From a clinical perspective, in individuals subjected to chronic or traumatic stress, neurogenesis in the hippocampus is impaired along with other brain areas involved in the evaluation and regulation of emotions, which would lead to the development of affective disorders. 28 Importantly, hippocampal neurogenesis is also modulated by physical activity. In 1999, exercise was elegantly shown to increase cell proliferation (∼ 50%) in the hippocampal area of adult mice. 29 Interestingly, recent studies suggest that exercise, when practiced in moderation and supervised by a qualified professional, not only serves as an effective method for improving physical health, but can also lead to an improvement in brain function and, therefore, act as a preventive and protective measure against numerous neurological and mental diseases. 30 In parallel, numerous studies have shown that excessive alcohol consumption causes harm of the CNS, with the hippocampus being the central target of its neurotoxic effects. 31 In this regard, several studies have shown that acute and chronic treatment with ethanol reduces hippocampal neurogenesis in rats. 32 Although it is reported that this decrease in neurogenesis must be related to the associated cognitive deficit with excessive alcohol consumption, a compensatory increase in neurogenesis during abstinence may have a direct impact on cognitive recovery. 31 32 In general, neurogenesis is currently thought to promote adaptability in response to environmental changes but is also considered an effective process for repairing neuronal networks after CNS injury or in CNS disease. 33

## HOW PESTICIDES CAN AFFECT NEUROGENESIS

In Brazil, in 2020, we had a total of 83,396,004 ha of cultivated land, an increase of 27.6% compared with 2010. 34 In parallel, the pesticide reports presented by the Brazilian Institute of the Environment (IBAMA, in the Portuguese acronym) clearly showed that between 2010 and 2020 the amount of pesticides sold in Brazil increased by 78.3%; additionally, 384,501.28 tons of active ingredients were sold in 2010, and 685,745.68 in 2020. 33 Therefore, we can state that the amount of pesticides sold in Brazil increased approximately 3-fold in comparison with the growth of cultivated areas in the country between 2010 and 2020. 34 According to data from the National Health Surveillance Agency (ANVISA, in the Portuguese acronym) from 2020, of the total active ingredients of pesticides (504 in total) that were registered for use in the country, 397 were industrially produced chemicals, 146 of which have no marketing and no approved use in Europe. 34 35 In general, the Brazilian pesticide market has grown rapidly and alarmingly over the last decade, placing Brazil first in the world ranking of pesticide consumption. 35

According to these arguments, it is well known that pesticides have serious effects not only on the environment but also on human health. In this regard, we confirm the current proposal that the use of pesticides in our country must be considered a public health emergency, given the size of the population living in and around pesticide factories, in agriculture, in nearby areas, and to all of us who are consumers of contaminated food. 35 In fact, epidemiological studies refer to some acute and chronic health effects of pesticide exposure, including dermatological, visual, auditory, respiratory, gastrointestinal, cardiovascular, fertility, carcinogenic and neurological. 36 With regard to the CNS, important studies have found that some families of pesticides (e.g., carbamates, organochlorines, and organophosphates) can cause severe damage to the CNS and are considered potential risk factors for the development of neurodegenerative diseases. 36 For example, 5 to 10 years of exposure to pesticides have been described to be associated with a 5 to 11% increased risk of developing Parkinson disease. 36 At the same time, several pesticides indirectly produce harmful neurological effects and unbalance the cellular mechanisms that maintain the metabolic activity of the CNS. 37 In addition, farmers exposed to pesticides are more susceptible to anxiety and depression. 38 39 From a pathophysiological point of view, it is likely that changes in synaptic structure and function play a fundamental role in the development of these neuropsychiatric conditions. 38 39 Therefore, the process of neurogenesis takes on particular importance in this scenario.

In the last decade, important studies have demonstrated that pesticides, mainly herbicides and insecticides exhibiting adverse effects on brain neuroplasticity, inhibit the formation of new neurons in the hippocampus. 40 For example, an interesting study evaluated the effects of exposure to permethrin (synthetic compound used in insecticides, repellents and acaricides) in laboratory rats for a period of 4 weeks, demonstrating a clear reduction in hippocampal volume and multiple cellular changes such as partial loss of neurons, inflammation of the brain parenchyma, and reduced neurogenesis. 41 In parallel, another study assessed the effects of administration the organophosphate chlorpyrifos (insecticide) over a period of 10 days (1 dose per day) on the morphometry of the hippocampus in laboratory mice. 42 The authors found compromised integrity of synapses and evident reduction in hippocampal neurogenesis, suggesting the occurrence of an early neurotoxic effect caused by organophosphates. 42 Furthermore, several experimental studies have shown that rotenone, an odorless chemical substance used as an insecticide, causes adverse effects on neurogenesis, brain electrical activity, and behavioral changes in in vivo and in vitro studies. 43 At the same time, other authors reviewed the effects of exposure to the insecticide deltamethrin for a period of 60 days on the behavior and brain plasticity of laboratory mice. 44 The authors noted a deficit in learning and memory and a significant reduction in neurogenesis in the hippocampus (37%) of animals treated with pesticides, suggesting that the changes promoted by pesticides in hippocampal plasticity directly influence cerebral information processing. 45 Simultaneously, several studies have revealed that paraquat, an ammonium compound used as herbicide, reduces neurogenesis and directly affects the survival and fate of new neurons generated in the hippocampus. 46

**Figure 1:**
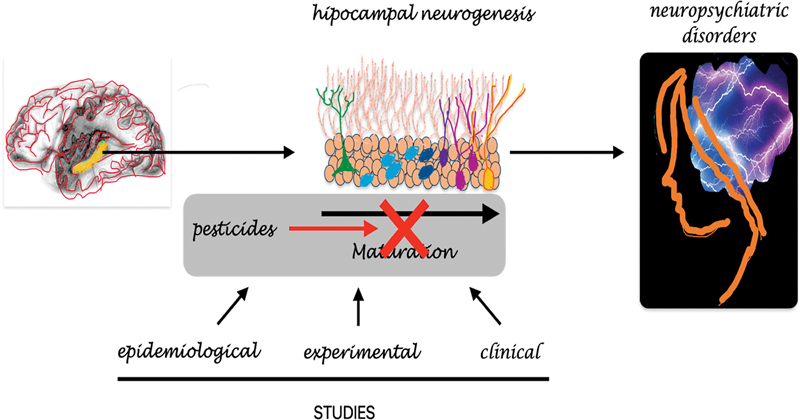
The serious effects of pesticides on human health, particularly on the brain, damaging hippocampal neurogenesis, require in-depth epidemiological, experimental, and clinical studies.

In conclusion, studies of neurogenesis in specific areas of the adult brain have promoted advances in several areas of biomedical research. In this sense, the impairment of hippocampal neurogenesis caused by pesticides is associated with the decline of neuronal and cognitive functions and the possible development of neuropsychiatric disorders Figure 1 . Finally, this scenario shows the importance of new experimental, epidemiological, and clinical studies to accurately determine the effects of pesticides on human health, which still poses challenges for medicine.
